# Lentiviral gene transfer into human and murine hematopoietic stem cells: size matters

**DOI:** 10.1186/s13104-016-2118-z

**Published:** 2016-06-16

**Authors:** Kirsten Canté-Barrett, Rui D. Mendes, Willem K. Smits, Yvette M. van Helsdingen-van Wijk, Rob Pieters, Jules P. P. Meijerink

**Affiliations:** Department of Pediatric Oncology/Hematology, Erasmus MC Rotterdam-Sophia Children’s Hospital, Wytemaweg 80, 3015 CN Rotterdam, The Netherlands; Princess Máxima Center of Pediatric Oncology, Utrecht, The Netherlands

## Abstract

Contemporary biomedical research increasingly depends on techniques to induce or to inhibit expression of genes in hematopoietic stem cells (HSCs) or other primary cells to assess their roles on cellular processes including differentiation, apoptosis and migration. Surprisingly little information is available to optimize lentiviral transduction of HSCs. We have therefore carefully optimized transduction of murine and human HSCs by optimizing vector design, serum-free virus production and virus quantitation. We conclude that the viral RNA length, even in relatively small vectors, is an important factor affecting the lentiviral gene transfer on the level of both the virus production and the cellular transduction efficiency. Efficient transfer of large gene sequences into difficult-to-transduce primary cells will benefit from reducing the lentiviral construct size.

**Electronic supplementary material:**

The online version of this article (doi:10.1186/s13104-016-2118-z) contains supplementary material, which is available to authorized users.

## Background

Human immunodeficiency virus-based lentiviral gene transfer has been embraced in contemporary laboratory practice as an efficient procedure to shuttle gene-encoding RNA molecules into target cells, where they are reverse-transcribed and integrated into the host genome. Third-generation lentiviral vector systems have proven to be safe methods in gene therapy with very low risks of ongoing integrations in the host genome or generation of replication-competent viral particles [1, 2]. Lentiviruses infect both dividing and non-dividing cells, making them ideally suited to transduce human and murine hematopoietic stem cells (HSCs) [3–6]. Many fields of research often require lentiviral constructs that drive gene expression from a promoter as well as a fluorescent reporter expressed from an internal ribosomal entry site or secondary promoter. The virus production (tested for vectors that encode viral RNA ranging from 4 to 7.5 kb in length) [7] and efficiency to transduce adherent cell lines seems dependent on the size of the lentiviral vector that encodes for the viral RNA. Vectors with viral RNA ranging from 5 to 9 kb generally tested decent, whereas those ranging from 10 to 18 kb transduced very poorly [8]. However, the nature of the target cell (cell line or primary cells) is crucial and most of the studies published to date have not addressed the transduction efficiency of primary cells. Additionally, it is important to control the production process of lentiviral particles (in HEK293T cells), and the quantitation method to determine the number of competent viral particles produced. Since little information is available regarding these variables and their effects on lentiviral transduction, we carefully optimized lentiviral transfer by optimizing vector design, serum-free virus production and quantitation, as well as by optimizing transduction of murine and human HSCs.

## Methods

### Cloning

**Fig. 1:**
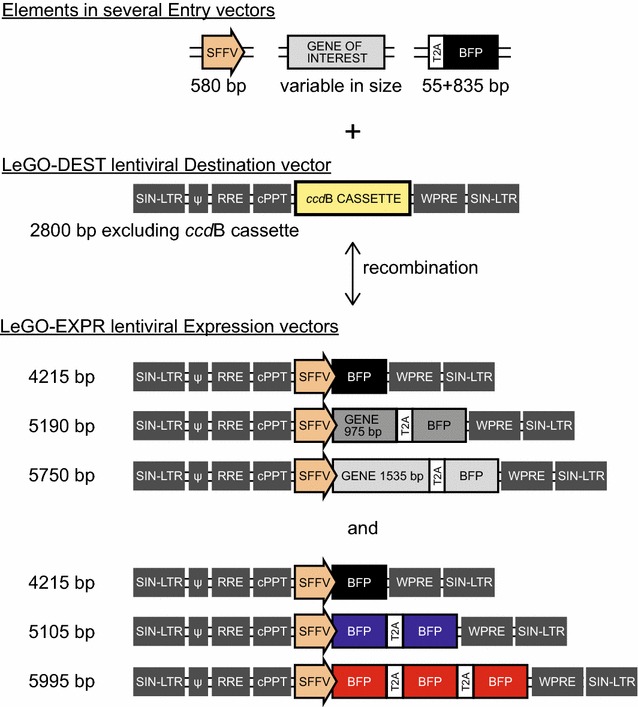
Schematic representation of the elements cloned into our Gateway compatible LeGO-DEST vector. The different elements and their sizes (bp) that were cloned into our Gateway compatible LeGO-DEST vector to generate lentiviral expression vectors of different sizes between SIN-LTRs: 4215, 5190 and 5750 bp. SFFV: Spleen Focus Forming Virus promoter, T2A: *Thosea asigna* virus 2A element, BFP: *blue* fluorescent protein. *Grey boxes* indicate lentiviral elements in LeGO-DEST

For fast, highly flexible and reliable cloning purposes, we have adapted the third-generation self-inactivating lentiviral LeGO-iC2 vector [9] into a Gateway compatible destination vector (LeGO-DEST) by replacing the *ApaI/PciI* 2 kb insert with the Gateway *ccd*B cassette (Life Technologies). We assembled lentiviral expression constructs in which we cloned a promoter, a gene, a *Thosea asigna* virus 2A (T2A) element [10], and the blue fluorescent protein reporter (mTagBFP). These, in combination with other lentiviral elements, are flanked by long terminal repeats and encode for the viral RNA. Upon translation, the gene and BFP moieties are efficiently separated by T2A cleavage in target cells (Fig. 1).

### Cell line

The human T cell leukemia cell line JURKAT (DSMZ, #ACC-282) identity was confirmed by DNA fingerprinting and cells were regularly tested for mycoplasma contamination.

### Virus production, concentration and quantification

We optimized HEK293T transfection in DMEM supplemented with 10 % serum using X-tremeGENE HP DNA Transfection Reagent (Roche, #06 366 236 001) to produce vesicular stomatitis virus-G pseudotyped virus particles without the addition of serum (low-serum Opti-MEM I with Glutamax: Life Technologies, #51985-026), in batches of 40 ml (harvest twice, with 24-h intervals, from two confluent 14-cm dishes starting 2 days after transfection, see Additional file 1). Low serum levels minimize the risk of premature differentiation of HSCs that are subjected to lentiviral transduction. Furthermore, fetal bovine serum can influence transduction [11], and we found that increasing amounts of serum (ranging from 0 to 10 %) negatively affects transduction rates of human HSCs, perhaps due to the aggregation of virus particles in the presence of serum proteins (data not shown). In relation to the quantitation of intact viral particles, the commonly used p24 protein ELISA method overestimates the number of functional viruses by the detection of incomplete, transduction-deficient viral particles as well as soluble p24 protein in the production medium [12]. Quantitative RT-PCR of encapsulated viral RNA particles is a valuable alternative. We therefore calculate the number of viral particles that we produce by quantification of viral RNA copies using RT-qPCR (two RNA copies per viral particle, see Additional file 1). RT-qPCR is performed using primers flanking the cPPT region: 5′-AGGTGGAGAGAGAGACAGAGAC-3′ and 5′-CTCTGCTGTCCCTGTAATAAAC-3′.

### Human CD34^+^ HSC transduction

Human CD34^+^ HSCs were positively selected from umbilical cordblood (Miltenyi Biotec, #130-100-453) and stimulated for 16-20 h at a concentration of 1x10^6^ cells/ml in X-VIVO 10 (Lonza, #BE04-743Q) supplemented with 50 ng/ml rhSCF (R&D, #255-SC), 20 ng/ml rhTPO (R&D, #288-TP/CF) and 50 ng/ml rhFlt3L (Miltenyi Biotec, #130-093-855). Prior to transduction, add protamine sulfate (Sigma, #P4020-1G) to the cells to a final concentration of 4 µg/ml and pipet the concentrated virus (IVSS VIVASPIN 20 centrifugation concentration columns, Sartorius AG, Sigma-Aldrich, #Z614653-48EA) into a 50 µg/ml retronectin (r-Fibronectin CH-296: TaKaRa, #T100A)-coated 96-well plate (Falcon, #351172). Add HSCs on top of the virus to a final volume of 200 µl/well and mix by gently tapping the plate. Spinoculation: centrifuge the cells in the virus-containing medium at 1800 rpm, 32 °C for 1 h. Incubate the transduced cells at 37 °C, 5 % CO_2_ for 24 h before further use.

## Results

**Fig. 2:**
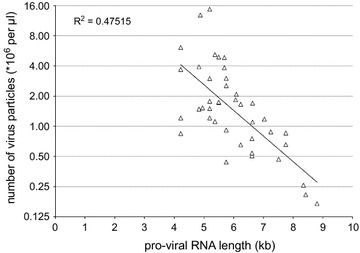
Relationship between the viral RNA length and production of lentiviral particles. The quantity of lentiviral particles that are produced (calculated using RT-qPCR) plotted as a function of the viral RNA length (kb). Each point in the *plot* represents an individual virus batch, each time produced using the pLEGO-DEST backbone and the same transfection method and producer cell line (see Additional file 1: Supplementary Protocol). The viral RNA length is the length of the sequence flanked by the 5′ and 3′ SIN-LTRs in the lentiviral expression vector (see Fig. 1)

**Fig. 3:**
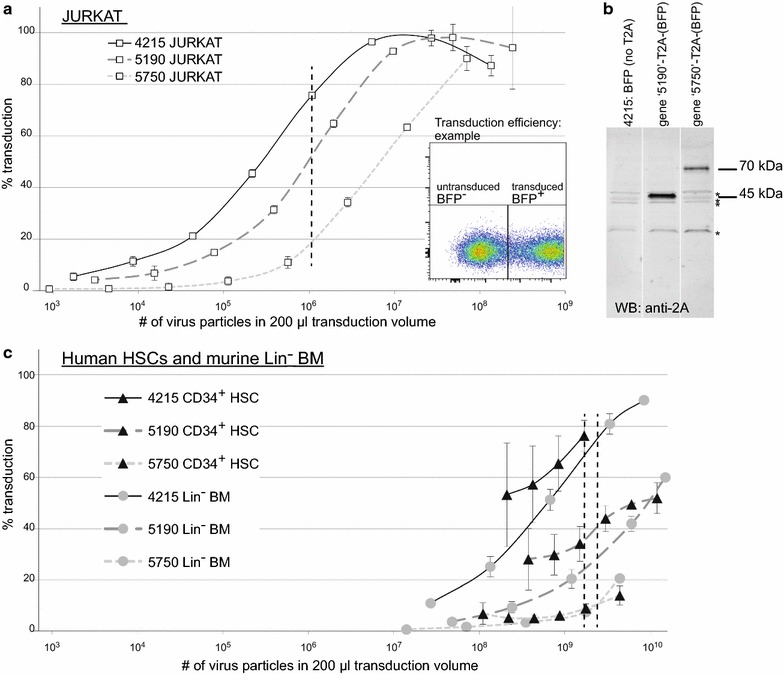
Transduction efficiency of JURKAT, CD34^+^ human HSCs and murine Lin^−^ BM with lentiviral vectors of varying sizes. Transduction efficiency of different cell types as a function of the amount of virus particles, measured 4–6 days after transduction and expressed as the BFP^+^ percentage of the viable cells. Lentiviruses with three different viral RNA sizes (length between SIN-LTRs) are compared: 4215 (*solid black lines*), 5190 (*dark-grey dashed lines*) and 5750 bp (*light-grey dashed lines*). **a** Triplicate transductions ± SD of JURKAT cells using serial dilutions of each lentivirus, representative of one of three individually produced virus batches. *Inset*: example flow cytometry plot displaying the BFP^+^ transduced fraction. **b** Western blot probed with anti-2A antibody. Total lysates expressing the T2A-tagged proteins of the 4215 bp (BFP only, no T2A), 5190 and 5750 bp constructs, separated from BFP. (*asterisk*): non-specific signals. **c** Triplicate transductions ± SD of human CD34^+^ HSCs (*solid triangles*) and murine Lin^−^ BM (*solid circles*) with the three lentiviruses

We set out to relate the length of the viral RNA to the efficiency of virus production as well as to the potency of these viral batches to transduce the T-cell acute lymphoblastic leukemia line JURKAT. Our optimized lentiviral particle production consistently leads to near equal yields for consecutive batches produced from the same lentiviral construct (average variation of 4.3 fold (range 1.3–12 fold) for repetitive viral batches from 10 different constructs). We observed an inverse exponential correlation between the length of the viral RNA encoded by the construct and the number of viral particles produced by HEK293T cells (Fig. 2). RT-qPCR in combination with functional titration experiments on a cell line reliably determines the number of viral particles required to efficiently transduce target cells. We investigated transduction efficiencies for virus batches of three different viral RNA lengths: 4215, 5190 and 5750 bases. For each construct, three independent viral batches were produced. Transduction experiments using serial dilutions of each viral batch were performed on JURKAT cells. Four to six days after transduction, the percentage of BFP-positive (transduced) cells was determined (Fig. 3a, representative curves for three independently produced virus batches of each construct). While the intensity of BFP signal was highest for the virus with the shortest viral RNA and lower for longer constructs, in each case the transduced population was easily distinguished from non-transduced cells (inset in Fig. 3a). The gene of interest encodes a protein with the C-terminal T2A that separates it from the BFP (Fig. 3b). The percentage of transduced JURKAT cells remained unchanged for over 3 weeks of culture (not shown), reflecting stable integration of the viral genome in the host DNA. All independently produced batches from the same lentiviral construct were virtually equally efficient to transduce JURKAT cells, indicating that the optimized production, quantitation and transduction procedures are very robust and reproducible. The transduction efficiency was highest for viruses with the shortest viral RNA sequence and became progressively lower with increasing length. This is also true for all other virus batches produced with viral RNA lengths varying from 4 to 9 kb. In general, more viral particles with longer viral RNAs seem to be required to achieve equal transduction percentages in target cells compared to viruses with shorter viral RNAs.

**Fig. 4:**
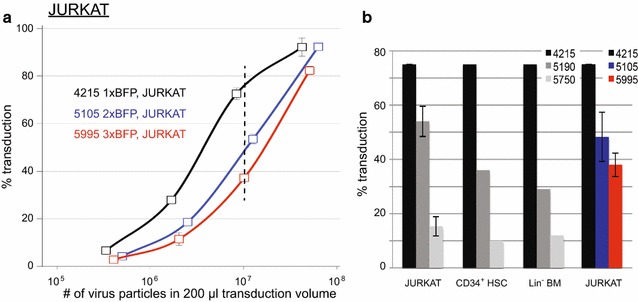
Transduction efficiency of JURKAT with independently generated and different lentiviral vectors of varying sizes. **a** Transduction efficiency of JURKAT cells as a function of the amount of virus particles, measured 4–6 days after transduction and expressed as the BFP^+^ percentage of the viable cells. Lentiviruses with three different viral RNA sizes (length between SIN-LTRs) are compared: 4215 (1xBFP; *black*), 5105 (2xBFP; *blue*) and 5995 bp (3xBFP; *red*). Triplicate transductions ± SD using serial dilutions of each lentivirus, representative of one of three individually produced virus batches. **b** Average transduction percentage of the different viruses, measured at the number of virus particles that yielded 75 % in the ‘1xBFP’ (4215 bp) vector (indicated by *dashed vertical lines* in Figs. 3 and 4a)

We then tested the efficiency of these viral batches to transduce primary human CD34^+^ HSCs and murine ‘lineage-negative’ bone marrow (Lin^−^ BM) cells. To achieve comparable transduction rates for JURKAT, human HSCs and mouse Lin^−^ BM, a thousand-fold more virus particles of the 4215 bp vector are required for the primary cells than JURKAT (Fig. 3c). This difference becomes even higher (up to 10^4^) for viruses with larger viral RNAs (5190 and 5750 bases). Thus, the viral RNA size moderately, albeit significantly, affects the transduction rate of a cell line such as JURKAT, but greatly influences transduction efficiencies of freshly isolated human HSCs and murine Lin^−^ BM cells. Transduced human HSCs cultured on stromal support cells retained an equal percentage of BFP-positive cells over the course of 3 weeks, indicating stable integration (data not shown). To investigate whether specific sequences can influence transduction efficiency, we generated new vectors harboring double or triple BFP sequences (5105 and 5995 bp, respectively; Fig. 1). These vectors only differ in size from the single BFP construct, but contain the same sequence. Also for these viruses, the transduction efficiency in JURKAT was highest for the shortest vector and reduced with increased length (Fig. 4).

## Discussion

We conclude that while larger viral RNA size negatively affects both virus production and transduction of target cells, other factors can also influence the transduction efficiency (e.g. sequence). This is evident from the observation that the 5750 bp vector (containing the 1535 bp gene) revealed lower transduction efficiency than the slightly larger triple BFP vector (5995 bp). The transduction efficiency of human or mouse stem cells decreases tremendously for viruses with viral RNAs approaching 6 kb or larger. Lentiviral vectors encoding smaller viral RNA sequences perform better and even a reduction of merely 600 bp (5750 versus 5190 bp) already improves transduction efficiency by more than threefold (Fig. 3). To produce more efficient lentiviruses, reducing the viral RNA backbone size by removal of non-essential sequences may be effective. Codon optimization alters the gene sequence without affecting the protein sequence and may also increase transduction efficiency. Additionally, the development of smaller reporter genes or complete removal of the reporter may further enhance the transduction efficiency. In the absence of a fluorescent reporter, integrated lentiviral constructs into the host genome or expression of lentiviral transgene mRNA can be quantified by qPCR [13, 14] or RT-qPCR [15], respectively. In conclusion, size reduction of lentiviral constructs will facilitate efficient transfer of large gene sequences into difficult-to-transduce primary cells and will be most helpful in many fields of (basic) research.

## Additional file

10.1186/s13104-016-2118-z Virus production and concentration, viral RNA isolation and RT-qPCR and human CD34^+^ HSC transduction.
