# Supplementary material for: Lentiviral gene transfer into human and murine hematopoietic stem cells: size matters
Source: BMC Res Notes. 2016 Jun 16;9:312. doi: 10.1186/s13104-016-2118-z (PMC4910193; doi:10.1186/s13104-016-2118-z)
Supplement: Supplementary file 1 — 10.1186/s13104-016-2118-z Virus production and concentration, viral RNA isolation and RT-qPCR and human CD34+ HSC transduction. [file 13104_2016_2118_MOESM1_ESM.docx]

**Supplementary Protocols: virus production and concentration, viral RNA isolation and RT-qPCR, and human CD34^+^ HSC transduction.**

**Reagents**

X-tremeGENE HP DNA Transfection Reagent: Roche *Cat.* #06 366 236 001

Opti-MEM I with Glutamax: Life Technologies *Cat.* #51985-026

0.45 µm filter: Minisart *Cat.* #17598 or equivalent

IVSS VIVASPIN 20 centrifugation concentration column: Sartorius AG, Sigma-Aldrich *Cat.* #Z614653-48EA

Retronectin: r-Fibronectin CH-296: TaKaRa *Cat.* #T100A

Non-tissue culture treated plates: Falcon (96 wp) *Cat.* #351172

X-VIVO 10 medium: Lonza *Cat.* #BE04-743Q

Recombinant human stem cell factor (rhSCF): R&D *Cat.* #255-SC

Recombinant human thrombopoietin (rhTPO): R&D *Cat.* #288-TP/CF

Recombinant human FLT3 ligand (rhFlt3L): Miltenyi Biotec *Cat.* #130-093-855

Protamine Sulfate salt from Salmon grade X: Sigma *Cat.* #P4020-1G

**Virus production and concentration (biosafety level 2)**

1. For a 14 cm dish, plate ~8x10^6^ HEK293T cells in 15 ml DMEM + 10% FCS and grow O/N to near confluency
2. Mix lentiviral vector DNA (25 µg), VSV-G (3.7 µg), RRE (GAG/POL; 5 µg), REV (3.1 µg) and serum-free Opti-MEM (4 ml)
3. Add X-tremeGENE HP DNA Transfection Reagent (37 µl) to the DNA mix
4. Incubate at room temperature for 15 minutes
5. In a drop-wise manner: add the transfection mixture to the cells
6. Incubate cells at 37ºC, 5%CO_2_ for 16-20 hours
7. Remove DMEM, wash cells once with pre-warmed PBS, and add 11 ml of pre-warmed serum-free Opti-MEM
8. Incubate cells at 37ºC, 5%CO_2_ for 24 hours
9. Collect the first batch of virus-containing medium and pass through a 0.45 µm filter
10. Concentrate roughly 40-fold (for example: 2 plates: concentrate 20 ml to 0.5 ml) using a centrifugation concentration column according to the manufacturer’s recommendations and keep at 4ºC
11. Add 11 ml of fresh pre-warmed serum-free Opti-MEM to the cells and incubate at 37ºC, 5%CO_2_ for another 24 hours
12. Collect the second batch of virus-medium and repeat steps 9 and 10
13. Combine both batches and determine the concentration factor
14. Take 2 µl of the concentrated virus for viral RNA isolation in step 17
15. Aliquot virus and use fresh or store at -80ºC

**Viral RNA isolation and RT-qPCR**

1. To determine the number of viral particles by PCR quantification, make a serial dilution of a viral vector DNA plasmid with a known concentration
2. Trizol isolation of viral RNA is performed according to the manufacturer’s procedure
3. RT-qPCR is performed using primers flanking the cPPT region: 5’-AGGTGGAGAGAGAGACAGAGAC-3’ and 5’-CTCTGCTGTCCCTGTAATAAAC-3’

**Retronectin-coated plates**

1. Coat retronectin at a concentration of 50 µg/ml in PBS in non-tissue culture treated plates at 4ºC (overnight)
2. Before using retronectin-coated plate: remove retronectin, add 2%BSA/PBS, incubate at 37ºC for 30 minutes, and wash twice with PBS

**Human CD34^+^ HSC O/N stimulation**

1. Resuspend HSCs to concentration of 0.5x10^6^/ml in serum-free X-VIVO 10 supplemented with 50 ng/ml rhSCF, 20 ng/ml rhTPO and 50 ng/ml rhFlt3L
2. Incubate the cells at 37ºC, 5%CO_2_ for 16-20 hours

**Human CD34^+^ HSC transduction (96 well-plate) (biosafety level 2)**

1. Collect HSCs and centrifuge to adjust to a concentration of 1x10^6^/ml, keeping them in the same medium with cytokines
2. Add protamine sulfate to the cells to a final concentration of 4 µg/ml
3. Pipet concentrated virus into retronectin-coated (96-well) plate
4. Add HSCs on top of the virus to a final volume of 200 µl/well
5. Mix by gently tapping the plate
6. Spinoculation: centrifuge at 1800 rpm, 32ºC for 1 hour
7. Incubate the transduced cells at 37ºC, 5%CO_2_ for 24 hours before further use
